# *hTERT* Epigenetics Provides New Perspectives for Diagnosis and Evidence-Based Guidance of Chemotherapy in Cancer

**DOI:** 10.3390/ijms25137331

**Published:** 2024-07-04

**Authors:** Simeon Santourlidis, Marcos J. Araúzo-Bravo, Robert T. Brodell, Mohamed Hassan, Marcelo L. Bendhack

**Affiliations:** 1 Institute of Transplantation Diagnostics and Cell Therapeutics, Medical Faculty, Heinrich Heine University Duesseldorf, Moorenstr. 5, 40225 Duesseldorf, Germany; 2 Group of Computational Biology and Systems Biomedicine, Biodonostia Health Research Institute, 20014 San Sebastián, Spain; mararabra@yahoo.co.uk; 3 Ikerbasque, Basque Foundation for Science, 48013 Bilbao, Spain; 4 Department of Cell Biology and Histology, Faculty of Medicine and Nursing, University of Basque Country (UPV/EHU), 48940 Leioa, Spain; 5 Department of Pathology, University of Mississippi Medical Center, Jackson, MS 39216, USA; rbrodell@umc.edu; 6 Institut National de la Santé et de la Recherche Médicale, 67000 Strasbourg, France; dr.hassan@gmx.de; 7 Department of Surgery, Tulane University School of Medicine, New Orleans, LA 70112, USA; 8 Department of Urology, Red Cross University Hospital, Positivo University, Rua Mauá 1111, Curitiba 80030-200, Brazil; marcelo@uro-onco.net

**Keywords:** *hTERT*, CpG island, DNA methylation, MSPCR, chemotherapy

## Abstract

Strong epigenetic pan-cancer biomarkers are required to meet several current, urgent clinical needs and to further improve the present chemotherapeutic standard. We have concentrated on the investigation of epigenetic alteration of the *hTERT* gene, which is frequently epigenetically dysregulated in a number of cancers in specific developmental stages. Distinct DNA methylation profiles were identified in our data on early urothelial cancer. An efficient *EpihTERT* assay could be developed utilizing suitable combinations with sequence-dependent thermodynamic parameters to distinguish between differentially methylated states. We infer from this data set, the epigenetic context, and the related literature that a CpG-rich, 2800 bp region, a prominent CpG island, surrounding the transcription start of the *hTERT* gene is the crucial epigenetic zone for the development of a potent biomarker. In order to accurately describe this region, we have named it “Acheron” (Ἀχέρων). In Greek mythology, this is the river of woe and misery and the path to the underworld. Exploitation of the DNA methylation profiles focused on this region, e.g., idiolocal normalized Methylation Specific PCR (IDLN-MSP), opens up a wide range of new possibilities for diagnosis, determination of prognosis, follow-up, and detection of residual disease. It may also have broad implications for the choice of chemotherapy.

## 1. Introduction

The renowned German chemist Paul Ehrlich coined the term “chemotherapy” in the early 1900s [1]. He used this phrase to describe the method by which chemicals, such as aniline dyes and the earliest crude alkylating agents, could be used to cure illnesses, including cancer [1]. Interestingly, he did not think his endeavor would be fruitful [1]. The first reports from World War I and II, indicating that troops exposed to mustard gas had altered bone marrow and lymph nodes, opened the door for the widespread use of nitrogen mustard on lymphoma patients in the United States after 1946. In fact, this therapeutic approach produced noticeable remissions; however, they were often fleeting [1]. Of course, mustard gases are DNA-alkylating substances that have harmful mutagenic and carcinogenic effects [2]. Thus, the concept of using chemicals to treat cancer was still highly controversial.

In the middle 1950s, Charles Heidelberger and colleagues at the University of Wisconsin developed 5-fluorouracil (5-FU) [3], a cytotoxic agent that acts by misincorporating fluoronucleotides into RNA and DNA [3], mainly by inhibiting the nucleotide synthetic enzyme thymidylate synthase (TS) [4]. TS inhibition causes imbalances of the dATP/dTTP ratio, which result in lethal DNA damage by disrupting DNA synthesis and repair [3]. It is still routinely used today to treat a variety of cancers, including liver, stomach, anal, pancreatic, oesophageal, cervical, bladder, and breast cancer [5]. It remains the primary chemotherapeutic treatment for colorectal cancer and can be used topically to treat premalignant actinic keratoses [6]. It is frequently used in conjunction with other cancer medications. The overall response rate for advanced colorectal cancer treated with 5-FU alone is 10–15%, while the response rate when 5-FU is combined with additional anti-tumor medicines is 40–50% [4]. Its drawbacks include systemic toxicity, the development of resistance, and a lack of efficacy and selectivity [6]. Cardiotoxicity is a serious side effect of 5-FU medication that affects cancer patients. It has been reported to occur in 1–20% of cases, and there have been cases of cardiac arrest or sudden death during 5-FU treatment [7].

Cisplatin is another example of a commonly used chemotherapy medication that has been used for 45 years in cancer treatment. It exhibits therapeutic efficacy against lung, cervical, breast, prostate, and head and neck cancers [8]. It is one of the most widely used treatments for bladder cancer, and is most effective, particularly for patients with testicular germ cell tumors or ovarian cancers [8]. The combination of surgery and cisplatin-based chemotherapy has resulted in a cure rate of >90% in patients with testicular cancer, although some patients become refractory to chemotherapy or have a late relapse [9]. The primary mechanism of action is its interaction with DNA, resulting in lesions that cause structural disruptions of the DNA molecule. These disruptions can either cause cell cycle arrest or initiate apoptosis, ultimately leading to cell death [8]. Its administration is associated with myriad adverse effects, resulting in complications such as vomiting, gastrointestinal disorders, and toxic manifestations affecting multiple organs and systems [10]. Nephrotoxicity, neurotoxicity, and ototoxicity are frequently observed [10].

It is noted that evidence-based science did not provide the foundation for this early chemotherapy research. Instead, these treatments resulted from coincidental observations and a confluence of audacious presumptions. Crucially, it is quite clear that these cytotoxic agents harm normal cells as well as cancer cells, with far-reaching negative consequences impacting morbidity and mortality—a phenomenon that cannot yet be predicted.

There is an urgent need for research and development to alleviate this situation. While patients with lung cancer often have molecular testing to determine which treatment is most likely to produce a remission, for many other patients’ treatment is based solely on the pathologic diagnosis with no way to determine if residual cancer remains. Therefore, concurrent chemotherapy treatment is used to eradicate any remaining tumor cells, and thus reduce the likelihood of a recurrence. The fear of a potential recurrence and the horrendous consequences that accompany it frequently lead to overtreatment [11], while in each individual case, the proportionality between benefit and harm remains concealed. For those patients who have no residual disease, unnecessary chemotherapy represents a toxic onslaught on their health. It is, therefore, critically important to identify suitable biomarkers to detect and monitor any residual disease and to provide evidence-based prognostic information upon which to base a recommendation for or against chemotherapy treatment [11,12]. New discoveries in the field of epigenetics, combined with recent discoveries on cell-free DNA (cfDNA), offer the potential for hope in achieving this goal [12].

First, early in the tumor’s development, necrotic and apoptotic cells of the main tumor release cfDNA into the circulation, according to pioneering research by Stroun et al. [13]. No cfDNA was detected in the plasma of 50 normal control subjects, and increased levels of cfDNA with neoplastic characteristics have been shown to differentiate patients with various forms of leukaemia, lung, prostate, pancreatic, kidney, and ovarian cancer from healthy people [13]. Specifically, cfDNA levels in plasma are notably raised in the presence of metastatic disease [13]. In Europe, these tumors account for 90% of all cancers [14].

Many studies have demonstrated the presence of tumor-specific epigenetic changes (e.g., CpG island hypermethylation) in cfDNA [14,15]. In addition, early on in a primary tumor’s formation, tumor cells seep into the blood. Both the genomic DNA, which came from freely circulating tumor cells, and the cfDNA are extremely promising sources for the development of powerful biomarkers [16]. Their covalently bound DNA methylation profiles may serve as epigenetic markers, identifying the specific cancer cells from which they are derived.

A recent, extensive study aimed at over 100,000 relevant methylation sites on cfDNA demonstrated that methylation marker analysis on cfDNA can precisely detect and pinpoint cancer [17]. The results from 6689 participants [2482 cancer (>50 cancer types), 4207 non-cancer] demonstrated, e.g., specificity of 99.3% in a validation set and stage I–III sensitivity of 67.3% in a pre-specified set of 12 cancer types (anus, bladder, colon/rectum, esophagus, head and neck, liver/bile-duct, lung, lymphoma, ovary, pancreas, plasma cell neoplasm, stomach) [17]. These account for ~63% of US cancer deaths annually [17]. Based on these and other study findings, the authors draw the conclusion that, by cfDNA sequencing using specific methylation profiles, it is possible to identify 50 cancer types in all phases of the disease [17].

A second study on differentially methylated regions (DMRs) unique to ovarian cancer (OC) found that cfDNA analysis in 61 cancer samples and 86 reference samples provided a sensitivity of 94.7% (95% CI: 85.4–98.9%) and a specificity of 88.7% [18]. The findings of this study collectively prove the validity and precision of cfDNA methylation markers for the identification and prognostic assessment of OC from plasma [18].

Nevertheless, these methods rely on mixing several epigenetically marked, specific cfDNA fragments, each of which has the potential to be differentially methylated in different tumor entities and different tumor stages of development. Since the individual parts of those cfDNA fragments with their characteristic DNA methylation profiles have not been validated for their constant occurrence in a specific, heterogenic tumor entity and distinct developmental stages, the clinical application of these differentially methylated marker panels requires additional work.

Indeed, clinicians fully understand the urgent need for unique malignancy biomarkers with a high level of specificity. Patients dread being told they have no clinical evidence of cancer, only to find out later that their disease is still progressing and that it might endanger their lives.

An ideal epigenetic biomarker for cancer—possibly even a strong pan-cancer biomarker—might be a single, reliable methylation change unique to cancer cells and absent from all healthy cells in the body, allowing it to probe the existence of a wide range of cancer entities. Further refinements in the technological advancement of this biomarker would be dependability, sensitivity, little invasiveness, and a quick and economical assay.

The development of cancer is characterized by unrestricted self-renewal. Malignant cells reactivate telomerase to stretch their telomeres and achieve cellular immortality, which is recognized as a “Hallmark of Cancer” [19]. In 1994, Jerry Shay and colleagues found telomerase activity in over 90% of human malignancies and cell lines [20]. None of the 50 normal somatic or benign tissues or the 22 normal somatic cell cultures had hTERT expression [20]. In 1998, the same group demonstrated that it was sufficient to induce cell immortalization in normal human cells by adding hTERT, the catalytic protein reverse transcriptase component of telomerase [21]. They conclude that telomerase is severely repressed in healthy human somatic tissues, while tumor formation depends on telomerase activity. Exceptions are the germ line and stem cells, which possess telomerase activity [22]. It has been suggested that novel diagnostic applications would result from this insight [20].

Accumulating evidence suggests that telomerase activity is regulated at the transcriptional level in cancer cells [22]. In normal human cells, transcriptional suppression of hTERT is the primary mechanism governing telomerase regulation [23]. In addition to genetic alterations, it is primarily epigenetic changes, in particular DNA methylation that is involved with gene activation in cancer cells [22]. Further comprehensive analyses of 18,430 samples from 31 different cancer types, including tumors (e.g., bladder-, prostate-, breast-, lung-, colon cancer, and melanoma), and non-neoplastic tissues have demonstrated the overriding importance of DNA methylation for the regulation of the *hTERT* gene [24]. The authors showed 73% of 6835 cancers had telomerase reverse transcriptase (TERT) expression and reported that 63% of TERT wild-type tumors in a core set that consisted of 473 T/N pairs expressed TERT, of which 91% showed promoter DNA methylation [24].

Meanwhile, there have been a plethora of publications reporting that DNA methylation of a certain region in the 5′-regulatory gene area plays a fundamental role in the regulation of the *hTERT* gene and that this is associated with tumor progression. In our own study, we demonstrated key details related to this mechanism, enhancing the prospect of developing tools focused on the clinical application of these findings.

Castelo-Branco et al. reported, in an analysis of 68 samples, the identification of a subset of five CpG sites upstream of the transcription start site, which were hypermethylated in all malignant pediatric brain tumors that expressed TERT but not in normal tissues that did not express TERT (*p* < 0.0001) [25]. In this study, their analyses confirmed that 25 CpG sites were not hypermethylated in eight samples of normal tissues and three of low-grade tumors, but were hypermethylated in 57 malignant samples (eight high-grade gliomas, 45 ependymomas, and four leukaemias [25]. They named this region the TERT Hypermethylated Oncological Region (THOR). These findings suggest that DNA-methylation-based markers could lead to the development of biomarkers for various cancers [25].

The same group later reported that THOR was hypermethylated in prostate cancer (PCa) when compared to paired benign tissues (*n* = 164, *p* < 0.0001) [26]. Furthermore, THOR hypermethylation, correlated with Gleason scores, was associated with tumor invasiveness (*p* = 0.0147) and was able to predict outcomes in the challenging Gleason 6 and 7 (3 + 4)) PCa (*p* = 0.007) [26]. Due to its high prevalence in more than 45% of all cancer types screened (1352 human tumors, 9 of 11 tumor types), THOR hypermethylation has been suggested to be a frequent telomerase-activating mechanism in hTERT-expressing tumor types, e.g., in cancers of the prostate, breast, blood, colon, skin, bladder, and brain. In this publication, the group defined THOR as a 433-bp genomic region that encompasses 52 CpG sites located immediately upstream of the TERT core promoter [27].

Furthermore, evidence has demonstrated that the TERT hypermethylated oncologic region predicts recurrence and survival in pancreatic cancer [28] is associated with higher TERT expression and higher-risk disease in non-muscle-invasive bladder cancers (NMIBC) [29]. Finally, THOR hypermethylation was suggested to be an important epigenetic mark in breast tumorigenesis [30].

Based on this broad evidence on the role of THOR in cancer, our team focused on identifying additional epigenetic features of this region [31]. First, we analyzed the detailed methylation pattern of the THOR. Our findings revealed a distinct DNA methylation profile that was present in superficial pTaLG urothelial cancers. Bisulfite genomic sequencing uncovered the detailed methylation profile comprising the methylation status of every single CpG position. By this sub-cloning sequencing method, one sequence represents the DNA methylation profile of one allele of a cancer cell. By analyzing, for example, thirty sequences, a high-resolution DNA methylation profile depicting the exact pattern of differential methylation at each CpG position can be generated, which represents the situation in the corresponding cancer cell population.

We observed single CpG dinucleotides that were completely methylated, while other CpG dinucleotides were completely unmethylated, and a few positions showed partial methylation. pT1HG urothelial tumors exhibited sequences in this region that had every single CpG methylated [31]. Evidence for that methylation starts at some more susceptible CpG positions and spreads from there into the neighborhood in the course of tumor progression. Completely methylated THOR was identified in advanced urothelial cancer specimens of pT3. A detailed analysis permitted our team to define the CpG dinucleotide positions that show constitutive methylation in cancer and the CpG positions that are more or less defiant of methylation. Hence, based on this knowledge, in combination with sequence-dependent thermodynamic features, it is possible to present ideal primers for Methylation Specific PCR (MSPCR). MSPCR can clearly and efficiently discriminate between the different methylation states, as demonstrated in our current publication for urothelial cancer [31]. The amplification efficiency of the hypomethylated sequences was around 300 times lower than that of the partly methylated sequences using a newly developed, relatively quantitative EpiTHOR *hTERT* assay. In turn, this was almost 70 times lower than that of the highly methylated sequences. Thus, using this method makes it possible to distinguish between these differentially methylated genomic states [31].

It is mentioned here that our current investigations reveal precise and comparable patterns of cancer cell methylation from prostate cancer tissue specimens and from urine-derived cfDNA from individuals with urothelial cancer. The approach we use, known as Methylation Specific PCR is economical, quick, and sensitive. For genetically aberrant DNA specimens, an idiolocal normalization of real-time Methylation-Specific PCR (IDLN-MSPCR) is used [32]. Here, the methylation-independent reference sequence utilized for normalization is chosen near the methylation-dependent target sequence. This ensures that the copy numbers of the reference and target sequences will be equal in samples of tumor DNA, which may have genetic variations. Otherwise, this could lead to imbalanced numbers of reference and/or target sequences, which would lead to incorrectly normalized real-time MSPCR results. This approach enables trustworthy comparative measurements of DNA methylation in the DNA of clinical samples with genetic imbalances [32]. Typically, populations of cancer cells have genetic heterogeneity. For example, numerous chromosomal abnormalities are associated with early high-risk (pT1) bladder cancer [33].

DNA methylation of CpG dinucleotides is a major factor in the division of the genome into transcriptionally competent, quiescent, and active regions. It also plays a part in the epigenetic regulation of cell fate and function that is specific to a given cell type [34]. The transcriptional start site of more than 60% of human genes is encircled by a “CpG-island,” which is a 0.4–2 kb long, CpG-rich region that is relevant for regulation depending on the DNA methylation state [34]. Expression is impacted by both partial and total methylation of the CpG dinucleotides inside these CpG islands [35]. Not every CpG inside a CpG island of a particular gene has the same capacity to affect expression as a function of methylation. When methylated, certain CpG dinucleotide locations influence gene silence more than others [35].

**Figure 1:**
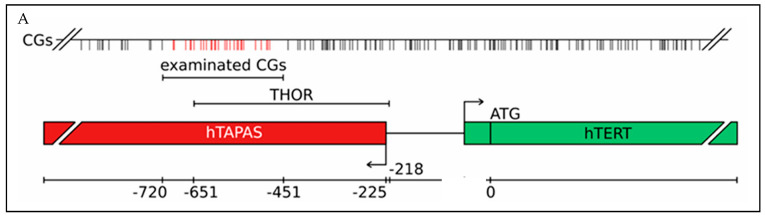

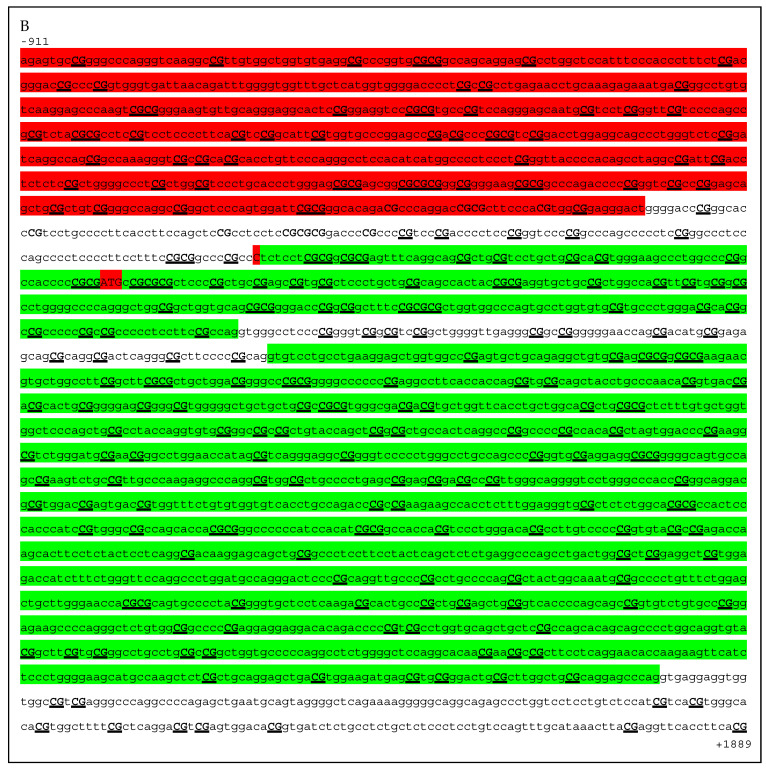
CpG island Acheron. (**A**) The examined CpG dinucleotides in early urothelial cancer samples led to the detailed methylation patterns and the EpiTHOR *hTERT* assay [31] is indicated by “examined CGs” and vertical red lines symbolizing CpGs. (**B**) (GRCh37/hg19): chr5, −1293216–1296015. This CpG rich Acheron region encompasses 2800 nucleotides surrounding the *hTERT* gene transcription start site. It has an average GC content of 71.3%. CpG dinucleotides are underlined. Characteristic sequence parts are highlighted by color: *hTAPAS* lncRNA (red)*,* C transcription start/ATG translation start (NC_000005.10 and NM_198253.3) (red), exon 1/2 (green).

However, we are facing in the case of *hTERT* in cancer one curious phenomenon: methylation of the 5′-regulatory region of *hTERT* correlates with *hTERT* expression in cancer [36]. Recent studies, among them our own investigation, suggest new epigenetic modes of *hTERT* gene activation [31,37]. The human telomerase reverse transcriptase (*hTERT*) 5′-region has a unique antisense transcript, indicating that the *hTERT* promoter is bidirectional. This 1.6 kb non-coding RNA is known as *hTERT* antisense promoter-associated (hTAPAS) RNA [37]. Both the nucleus and the cytoplasm include hTAPAS transcripts, which begin 167 nucleotides upstream of the *hTERT* transcription start site, Figure 1. In various cancer sample types, there is an inverse correlation between *hTERT* expression and *hTAPAS* expression [37]. Based on their findings, the authors suggested that this lncRNA negatively regulates *hTERT* expression, which is connected to telomere homeostasis and oncogenesis [37].

In our study, we demonstrated this inverse correlation in primary urothelial carcinoma samples and found that the CpG-rich THOR represents a proximal part of the CpG rich 5′-regulatory region of the hTAPAS transcript (Figure 1) [31]. We conclude that the methylation profile of this THOR mirrors the extent of the epigenetic impairment of *hTERT* expression control in early and advanced urothelial cancer. These findings suggest a new, straightforward explanation for the apparent paradoxical relation between methylation of the THOR and increased *hTERT* expression. According to this theory, dense DNA methylation at the THOR is located in the 5′-region of *hTAPAS*, which would repress the expression of this long, non-coding RNA [31]. This would alleviate *hTERT* repression by hTAPAS, leading to increased *hTERT* expression in spite of higher methylation in this upstream, distal region of the *hTERT* promoter. Thus, the prime target of epigenetic repression by THOR methylation is *hTAPAS*, and the effects on *hTERT* expression are primarily indirect. Specifically, dense DNA methylation of the *hTAPAS* CpG-island appears to contribute to *hTERT* re-expression in cancer [31].

In this context, the publication of Teisha J. Rowland et al. provides additional clarification. They suggested that, for the majority of cancers, TERT reactivation may be entirely epigenetic [38]. Across 23 different cancerous tissue types, using Bis-Seq data from 833 different cancer cell lines, they demonstrated that the DNA surrounding the *hTERT* transcription start is hypermethylated in the distal promoter region, while there is hypomethylation in the *hTERT* proximal promoter, flanking the transcription start site region [38]. In this proximal promoter region, methylation was allele-specific, and decreased methylation is associated with marks of active TERT transcription [38]. They concluded that TERT expression in cancer lines is canonical in respect to hypomethylation, despite the occurrence of an unusual upstream located hypermethylated region. Furthermore, they showed that this hypomethylated region is large and slightly more downstream than previously described, spanning −220 to +231 bp of the AUG [38]. This includes all, e.g., of TERT exon 1, and they remark that this may be a universal correlation and possibly a necessity for TERT expression in cancer cells [38].

Thus, it is evident that *hTERT* expression is widely induced in cancer, and this is based on a complex epigenetic regulation by DNA methylation of a large CpG-rich area flanking the transcription start. Differential methylation of THOR is a crucial component. Due to our own investigations and the evidence provided by the literature, we came to the conclusion that the detailed methylation profile of this whole CpG-rich region of a distinct cancer entity is a potentially versatile and valuable source for developing potent diagnostic and prognostic tools based on MSPCRs. This source is intended to provide us with distinct DNA methylation profiles that occur constitutively in a tumor entity and in a definable tumor stage. For instance, in our recently published study, we reported that such consistently occurring hypomethylations have been found for prostate cancer when we examined individual 60-bp-long CpG-rich sequences [39]. In our point of view, it will be necessary to define the detailed DNA methylation profile of this region for every relevant tumor entity and stage. We define this relevant DNA segment of 2800 nt length with a CG content of 71% that is located in (Chr5: 1,293,216–1,296,015, GRCh37/hg19). To ease the precision of reference, we decided to give this region the name Acheron (Ἀχέρων). This is of Greek mythological origin and refers to the river of woe and misery and the path to the underworld. This refers to both the distinct cancer cell-specific DNA methylation stream of this CpG island and the CpG island itself, which is evidently involved in *hTERT* activation and in the cancer cell immortality of several cancers.

We are now in the process of determining the exact DNA methylation pattern of the Acheron region for each relevant tumor entity. Likewise, we will assess this for early as well as later stages and for tissue adjacent to tumors. We need to additionally assess whether this DNA methylation of the Acheron region is already present in pathologically healthy surrounding tissue. This could be a predisposition for later carcinogenesis and recurrence after therapy, respectively.

This may inform the work of Slaughter et al. [40], who presented the idea of a field effect in cancer. This is commonly referred to as a field defect or field cancerization. Their studies of microscopic hyperplastic abnormalities of contiguous, benign tissue serve as evidence for this notion. They believe field cancerization is a significant contributing factor in cancer recurrence following therapy [40]. In fact, modern molecular biology tools have demonstrated molecular abnormalities in a variety of tissues that appear histologically normal. This includes the lung, breast, stomach, prostate, rectum, colon, and neck. These findings helped to prove that one of the key mechanisms underlying cancer’s multicentricity is the field effect [41]. Though the exact processes underpinning the field effect in cancer remain unclear, increasing molecular data suggest that genetically modified cells and changes in DNA methylation patterns are important factors [41]. Accordingly, it has been demonstrated that PCa is nearly invariably multi-focal [41], with aberrant cytomorphologic, genetic, epigenetic, and gene/protein expression in the histologically benign tissue around the tumor [42]. For example, Mehrota et al. used 159 biopsy cores from 37 prostatectomy samples and detected an epigenetic field effect for the genes APC, RARb2, and RASSF1A up to 3 mm from the malignant core in thee prostatectomy samples [43]. In this context, Acheron hypermethylation-based MSP tools, such as, e.g., the EpiTHOR *hTERT* assay [31] of multiple tissue samples representative of the whole gland appear promising in determining whether to use focal therapy (FT). FT is a minimally invasive method, e.g., High Intensity Focal Ultrasound (HIFU), Focal Cryotherapy, Irreversible Electroporation (IRE), Focal brachytherapy, Focal Laser Ablation (FLA), etc., which can produce excellent results in terms of safety and functional outcomes.

One of the global pioneers of high-intensity focused ultrasound therapy (HIFU) for prostate cancer is Professor Marcelo Bendhack [44]. He began his medical career in 1994 at the University Hospital (UKD) of the Heinrich Heine University, Duesseldorf, Germany. Since 2009, he has continued his academic activities at the University Hospital, Positivo University, Curitiba, Brazil. He has performed around 800 radical prostatectomies, and lately he has treated over 850 patients with PCa with HIFU. Publications on the subject attest to the positive oncological effects of HIFU [45]. However, a few of those patients experienced relapses (usually a second occurrence in the prostate). One common clinical question in the follow-up of patients after focal therapy is the variation in PSA (prostatic specific antigen) levels. Such results as PSA may indicate two possibilities: (a) usual PSA fluctuation (due to normal residual tissue) or (b) elevation of PSA through production by cancer cells. Such an assay like *EpihTERT* could definitely help physicians regarding decision-making about timing to indicate possible imaging examinations or complementary treatment. Here, a new possibility is seen to further improve the oncological outcomes.

A further possible clinical application is to use informative MSPCR Assays based on differential methylation of the Acheron sequence, like the EpiTHOR *hTERT* assay, to interrogate the mentioned distinct cancer cell-specific DNA methylation patterns from the cfDNA of the tumor surrounding body fluids. This is patient-friendly since it is minimally invasive at best and cost-effective. It has been documented that in healthy donors, cfDNA is released by cellular processes of apoptosis, necrosis, and secretion. Its concentration does not exceed 5–10 ng/mL [46]. It is shown that the main origins of this DNA fraction are white blood cells (55%), erythrocyte progenitors (30%), vascular endothelial cells (10%), and hepatocytes (1%) [47]. In addition, it has been demonstrated that the plasma of older people shows significantly higher levels of total cfDNA [47]. In cancer, additional cfDNA is released by apoptosis or necrosis of dying tumor cells or is shed by viable tumor cells [48]. Hence, the total cfDNA concentration may increase by up to 50-fold compared to healthy people. This depends on the type of cancer and the burden of the disease [46]. Interestingly, total cfDNA levels decrease after therapy or surgery for cancer [48].

Here, a plethora of possible clinical needs could be met by determining a specific Acheron methylation signature on cfDNA. These range from early detection, prognosis, follow-up, the detection of minimal residual disease, and supporting decision-making for or against chemotherapy. All of this could impact all the tumor entities mentioned. To come back to our introductory considerations on chemotherapy: An Acheron methylation signature or the absence of it, from, e.g., cfDNA and/or cellular DNA of blood, detected by normalized, sensitive, fast, and cost-effective MSPCR, has the potential to better determine which patients require chemotherapy following surgery. What did the ancient living Greeks do when they met the death river Acheron? It is conveyed that once the Greeks met the green/blue waters of this river, they immediately understood the sign and took all necessary measures to avoid this path that would otherwise lead them to death [49].

## 2. Conclusions

Revealing the detailed DNA methylation patterns of the whole Acheron region in all tumor entities and exploiting them by, e.g., MSPCR will open up a new broad perspective for the application of specific and potent epigenetic assays to efficiently address a plethora of urgent clinical needs, including the guidance of chemotherapy.
